# The effectiveness of Nurse Practitioners working at a GP cooperative: a study protocol

**DOI:** 10.1186/1471-2296-13-75

**Published:** 2012-08-07

**Authors:** Nancy Wijers, Lisette Schoonhoven, Paul Giesen, Hubertus Vrijhoef, Regi van der Burgt, Joke Mintjes, Michel Wensing, Miranda Laurant

**Affiliations:** 1 Scientific Institute for Quality of Healthcare, Radboud University Nijmegen Medical Centre, P.O. box 9101, Nijmegen 6500, HB, The Netherlands; 2 Faculty of Health Sciences, University of Southampton, Southampton, UK; 3 Scientific Center for Care and Welfare, Tilburg University, Tilburg, The Netherlands; 4 Foundation for Development of Quality Care in General Practice, Eindhoven, The Netherlands; 5 University of Applied Sciences, Nijmegen, The Netherlands

**Keywords:** Substitution, General practitioner, Nurse practitioner, Out of hours care, Quality, Safety, Costs

## Abstract

**Background:**

In many countries out-of-hours care faces serious challenges, including shortage of general practitioners, a high workload, reduced motivation to work out of hours, and increased demand for out-of-hours care. One response to these challenges is the introduction of nurse practitioner as doctor substitutes, in order to maintain the (high) accessibility and safety of out of hours care. Although nurse practitioners have proven to provide equally safe and efficient care during daytime primary care, it is unclear whether substitution is effective and efficient in the more complex out of hours primary care. This study aims to assess the effects of substitution of care from general practitioners to nurse practitioners in an out of hours primary care setting.

**Design:**

A quasi experimental study is undertaken at one “general practitioner cooperative” to offer out-of-hours care for 304.000 people in the South East of the Netherlands. In the experimental condition patient care is provided by a team of one nurse practitioner and four general practitioners; where the nurse practitioner replaces one general practitioner during one day of the weekend from 10 am to 5 pm. In the control condition patient care is provided by a team of five general practitioners during the other day of the weekend, also from 10 am to 5 pm. The study period last 15 months, from April 2011 till July 2012.

**Methods:**

Data will be collected on number of different outcomes using a range of methods. Our primary outcome is substitution of care. This is calculated using the number and characteristics of patients that have a consultation at the GP cooperative. We compare the number of patients seen by both professionals, type of complaints, resource utilization (e.g. prescription, tests, investigations, referrals) and waiting times in the experimental condition and control condition. This data is derived from patient electronic medical records. Secondary outcomes are: patient satisfaction; general practitioners workload; quality and safety of care and barriers and facilitators.

**Discussion:**

The study will provide evidence whether substitution of care in out-of-hours setting is safe and efficient and give insight into barriers and facilitators related to the introduction of nurse practitioners in out-of-hours setting.

**Trial registration:**

ClinicalTrials.gov ID NCT01388374

## Background

Many countries are facing challenges concerning the accessibility, efficiency and quality of out of hours care. To address these challenges, significant changes in health care systems were seen in different European countries in the last few years. Most of these changes, involve a shift from practices collaborating in small-scale call rotations to the development of large scale organisations [1-3]. In the Netherlands out of hours care is organised in large scale General Practitioner Cooperatives (GPCs, see Table1), which were first established around the year 2000 [4]. Nowadays, there are 128 GPCs to cover out of hours care for almost all inhabitants of the Netherlands (approximately 16.7 million). Most of these GPCs are situated in or near a hospital with an Emergency Department (ED) [1,5]. 

**Table 1:** **Features of GP cooperatives (GPCs)**[4]

After-hours is from 5 p.m. to 8 a.m. daily and the entire weekend
Population includes 100 000 to 500 000 patients
Distances to GPCs are no more than 30 km
A GPC is usually situated near a hospital
Access through a single, regional telephone number is available
Telephone triage is conducted by nurses who are supervised by GPs
50 to 250 GPs are on call, with a mean 4 h of duties per week
A GPs shift is 6 to 8 h, with compensation of about €65/h
Per-shift GPs have different roles: home visits, center consultations, and telephone triage supervision
Drivers use identifiable GP cars that are fully equipped (e.g., oxygen, intravenous drip equipment, automated external defibrillator, and medication)
Information and communication technology support is available, including electronic patient files, online connection to the GP car, and sometimes connection with the electronic medical record in the GP daily practice

In spite of these changes and developments, primary out of hours care is still under pressure. A rising demand for (non-urgent) acute care, economic considerations, and the expected future shortage of GPs are important factors [1,6]. In addition, satisfaction among GPs is decreasing due to an increasing inappropriate demand for out of hours care and the demanding and aggressive behaviour of a number of patients who make use of this care [1,7]. In 2010, out of hours consultations increased with 39% compared with 2004. Almost half of the consultations were face to face consultations, resulting in a high number of patients visiting the GPC, especially during the weekend [8]. In addition, the EDs experience a rising demand for acute care as well. Most Dutch EDs are facing substantial numbers of self referred patients ranging from 25% to as high as 70% of all their in and out of hours demand [9-12]. These self referred patients present themselves often with low-complex and non-urgent complaints and could therefore also be seen in a primary care setting [11]. A study at a GPC situated at the ED of a hospital in the Netherlands showed that before the establishment of the GPC, 1592 out of 2199 patients (72%) utilized out of hours primary care at the primary care physician practices (PCPs). After the establishment of the GPC, 1990 out of 2278 patients (90%) utilized out of hours primary care instead of out of hours emergency care [13]. A Dutch study showed that medical costs for self referred patients at the ED are almost three times as high as for medical care provided by a GP during out of hours. A shift from ED care to GP care could therefore result in a remarkable cost reduction [14]. However, the rising demand for care in the acute setting and the shift from secondary to primary out of hours care also results in a high objective and subjective workload for GPs. It is expected that the pressure on GPs will increase even more in the next few years.

About 80% of the out of hours care is neither complex nor urgent from a medical perspective. It seems not necessary that these patients are seen by a physician [7]. A recent study [15] showed that NPs can substitute GPs in the management of patients with minor health problems during daytime primary care in a cost-effective way. The NPs acted independently in about 90% of the consultations and the quality of care they provided was comparable to care provided by the GP [15]. These findings have been confirmed by other research across the world. A systematic review of studies of doctor-nurse substitution in primary care showed that nurse led care did not result in appreciable differences between doctor and nurse care regarding health outcomes for patients, process of care, resource utilization or costs [16]. It is therefore anticipated that allocation of care during out of hours from GPs to NPs will positively contribute to the quality of care, improving accessibility and reducing the workload of GPs.

However, concerns have been expressed by some stakeholders regarding the introduction of NPs [17]. The expectation that total healthcare costs are reduced may be unfounded [16], NPs may lack crucial knowledge to provide safe healthcare for acute problems, and introduction of a new profession in primary care may result in fragmented healthcare delivery. In addition, studies on nurse involvement have mainly focused on daytime primary care and the challenges in out-of-hours care are crucially different. For instance, patients who contact the GPC are usually unknown, the health problems are more acute, and access to their medical record is limited or absent [18]. One of the main motives for patients to contact the GPC are concerns regarding their health [19].

### Objective

This study aims to assess the effects of substitution of care from general practitioners to nurse practitioners in an out of hours primary care setting.

## Methods

### Study design

The study is planned as a single-centre, non-blinded quasi-randomized trial with weekend days systematically allocated to either intervention or control condition. In the experimental condition patient care is provided by a team of one NP and four GPs. The NP replaces one GP during the weekend from 10 am to 5 pm. In the control condition patient care is provided by a team of five GPs on the other weekend day from 10 am to 5 pm (usual care). The intervention and control days rotate systematically every five weeks between Saturday and Sunday to reduce bias due to differences between the number of consultations and the difference in complaints presented on Saturday and Sunday. In addition, we collect data on a 15 month period preceding the observation period (April 2009 till June 2010) as historical control. The Medical Ethical Committee Arnhem-Nijmegen waived approval.

### Setting

**Figure 1:**
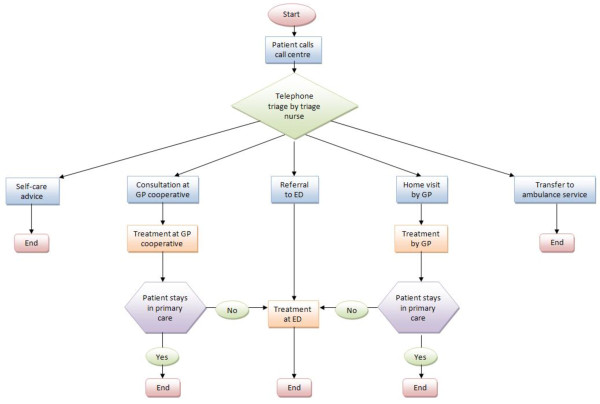
Patient flow for telephonic triage.

**Figure 2:**
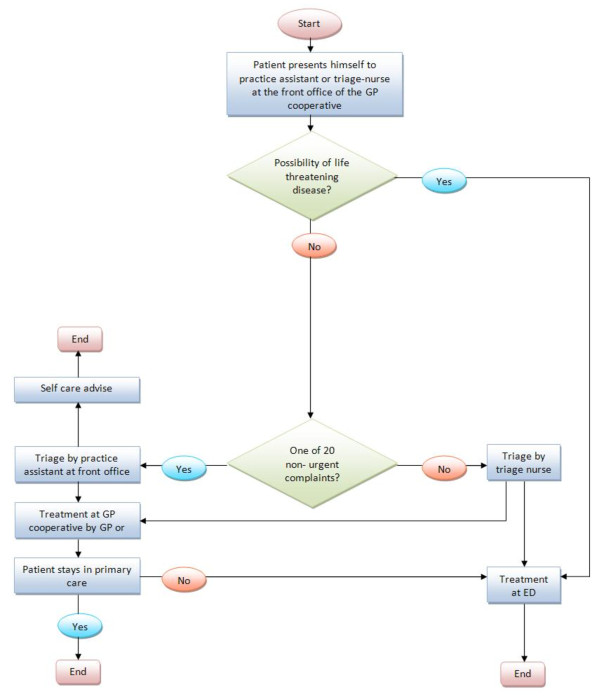
Patient flow for self referred patients.

The study was conducted at one large GPC in the South East of the Netherlands, situated within a hospital next to the ED. This GPC provides out of hours care for a population of approximately 304.000 people. In principle, patients in need for out of hours care contact the GPC by a single, regional telephone number. All calls come in at one call centre where the telephone triage is carried out by trained and qualified telephone triage nurses using the Netherlands Triage System (NTS) [20]. They assess the complaints, reason for calling, determine the urgency of the patient’s health problem and decide which action should be taken (i.e. self-care telephonic advise, a consultation at the GPC, a home visit or referral to the ED or ambulance service) [4,21,22]. From a total of 134.978 contacts in 2010 in this region, 57.9% were scheduled for an appointment at the GPC. To attend the GPC a telephonic contact is strictly recommended but many patients, approximately 20%, attend the GPC without an appointment for consultation. These self-referred patients present themselves directly to the practice assistants/triage nurse of the GPC with a medical concern. In those cases, the practice assistant or triage nurse performs the triage at the front office; assess the urgency of the patient’s health problem using the NTS and makes a decision about the appropriate action to be taken. (S)he can choose to give the patient self-care advice or to make an appointment for consultation at the GPC. (S)he also has the opportunity to refer the patient to the ED services since there is a formally regulated patient flow in conjunction with the ED. See Figure1 and 2 for patient flow. Both GPs and NPs have been trained to follow prevailing Dutch Clinical Practice Guidelines for General Practitioners, when applicable.

Patients are scheduled every ten minutes per professional by triage nurses who perform the telephone triage at the call centre or by practice assistants or triage nurses, at the front office of the GP cooperative, who perform the triage for self referred patients. The NP and GPs use the same diary. Patient allocation does not occur randomly since both the GPs and the NP choose their own patients from the common diary based on the presenting complaints. Random allocation of patients to NP or GP was not feasible as it would have interfered too much with daily routines. Current design represents daily practice more accurate. Since the NP does not have the full authority to prescribe medications, a GP is always available for consultation and to approve prescriptions.

### Study population

#### Patients

Patients with complaints of less urgent symptoms (urgency level U3 and U4, see Table2) that cannot wait until the next day as well as all self referred patients during the weekend between 10 am and 5 pm are included. The study focuses on patients who attended the GPC, regardless of who actually treats them – GP or NP. However, some patients groups are excluded from NP-care:

Patients under the age of 1 year;

Patients presenting themselves with psychiatric complaints;

Patients presenting themselves with abdominal pain, abdominal infections, chest pain or neck complaints, headache and dizziness.

**Table 2:** **NTS Urgency levels**[20]

Urgency level 1 (U1) – Life threatening:
Immediate action required, the vital functions are threatened or delaying treatment will cause serious and irreparable damage to the patient’s health.
Urgency level 2 (U2) – Emergent:
Vital functions are not (yet) in danger, but there is a fair change that the patient’s condition will soon deteriorate or delaying treatment will cause serious and irreparable damage to the patient’s health. Take action as soon as possible.
Urgency level 3 (U3) – Urgent:
Do not postpone too long. Treat within a few hours because of medical- or humane reasons.
Urgency level 4 (U4) – Non-urgent:
There is no pressure resulting from medical- or other grounds. Time and place of treatment should be discussed with the patient.
Urgency level 5 (U5):
A physical examination can wait until the next day.

Additional file 1 lists the complaints that NPs are qualified to diagnose and treat independently during out of hours care.

#### Nurse practitioners

Five qualified NPs were recruited. They are all registered nurses who completed a specific two year practice-oriented master training program: the Higher Professional Education Master’s Degree Advanced Nursing Practice (MANP)- Primary Care [23]. Their training included an academic course on managing common complaints in a primary care setting. During their educational training they were employed and trained in general practices and at the start of the intervention period (April 2011) they all had at least 5 years experience as NP in a day practice. The NPs received three half days of extra training in the diagnosis and treatment of eye disorders, musculoskeletal disorders (such as fractures, bruises and sprains) and wound care (suture) prior to the intervention period. These disorders are not very common in the day practices and extra training was therefore necessary. Furthermore, they had an introduction day at the GPC and they were present during one shift at the GPC with their GP supervisor. Characteristics of the professionals are described in Table3. 

**Table 3:** Characteristics of professionals

	**General practitioners (N = 138)**	**Nurse practitioners (N = 5)**	**Practice assistants (N = 9)**
Age in years (mean (SD))	49.4 (9.0)	40.4 (10.0)	39 (6.1)
Sex (% female)	39.9	100.0	100.0

#### General practitioners

One hundred and thirty eight (n = 138) GPs work at the GPC in Eindhoven. On average they are a member for 7.4 years (SD = 3.7) and have their own practice in the surrounding area. The GP practices are open during office hours between 8 am and 5 pm on weekdays.

#### Practice assistants/triage nurses

Ten practice assistants work at the entrance of the GPC. On average they have seven years (SD = 7.3) work experience at the GPC (range 3–10). These practice assistants work only at the GPC and not at the call centre.

## Measures

### Primary outcome

Our primary outcome is *substitution of care.* Substitution of care is calculated using the number of patients that have a consultation at the GP cooperative. We compare the number of characteristics patients seen by NPs and by GPs within the experimental condition and compare this with the number and characteristics of patients seen by the GPs in the control condition. Besides demographic patient data, such as gender and age, type of complaints (ICPC) and the urgency levels type of complaints (ICPC) will be included as patient characteristics. In addition, we compare GPs and NPs according to the variables waiting time and productivity (number of consultations). This data is derived from the patient electronic medical records.

### Secondary outcomes

#### Quality and safety of care

Quality and safety of care are measured by video/audio recording. In total, about 48 consultations are recorded, i.e. 24 consultations of the GP and 24 of the NP. Per professional approximately 6 consultations related to three common complaints are taped. Consultations skills of both professionals are assessed using the MAAS-Global instrument [24,25]. This validated instrument is widely used for communication assessments and was developed at Maastricht University for educational purposes [26]. Medical care of the GP and NP are measured using a checklist based on the Clinical Practice Guidelines for General Practitioners. Furthermore, patients with a recorded consultation are asked if the researcher can phone them approximately one week after the visit at the GPC in order to investigate if complications or misdiagnoses had occurred.

#### Patient satisfaction

Patient satisfaction is measured with the Consumer Quality Index (CQ-index). The CQ-index is the Dutch standard for measuring patient’s experiences of care [27]. Extra questions have been added to focus on specific topics, such as referral to the emergency department (ED), referral for X-ray or questions about possible adverse events or complications. The EQ-5D instrument [28] has been added to measure quality of life. In addition, extra questions about possible referral to the GP have been added for patients who had a consultation with the NP.

Prior to the intervention period and after approximately two, nine and fourteen months after the introduction of the NPs, a random selection of patients receive a letter by regular mail with the request to fill in a web-based questionnaire (CQ-index) about their experiences with the care received at the GPC. The letter includes information on how to fill in the questionnaire, a link to the web-based questionnaire, confidentiality of data, and a non-respondent form. Patients who do not want to participate in the study are asked to fill in this form and to report their reasons for not participating. If patients are not able to fill in the web-based questionnaire, they can ask for a written questionnaire send to them by regular mail, including a pre-paid return envelope. Up to two reminders are sent. The last reminder also includes a written questionnaire.

Baseline questionnaire invitations are only sent to a random selection of patient consulting a GP. Baseline measurement covered a random selection of 200 patients who consulted the GPs at the GPC during two consecutive weekends. At the three follow-up measurements a random selection of maximum 200 patients seen by the GPs during a five week period (control day) is selected. To ensure that we have sufficient numbers to answer our research questions, we send questionnaires to all patients seen by the NP during the same five week period (intervention days). For the measurement after two months after the introduction of the NPs, we send questionnaire to all 71 patients who consulted the NPs. The sample is a mix of patients with an appointment on Saturday or Sunday to avoid bias. It is expected that a relatively larger sample (approximately 100 NP patients per measurement) can be included at the final two measurements. There are no restrictions of the type of patients selected from the electronic medical records of the GPC, except that they should live in the Netherlands.

#### Subjective workload of professionals

Subjective workload of professionals working at the GPC is measured using the KwaliteitMeetSysteem” (KMS) questionnaire [29]. Prior to the intervention period and at the end of the intervention period all GPs and practice assistants working at GPC receive the questionnaire by e-mail. Up to two reminders are send. Non-relevant questions have been removed to shorten the questionnaire since we assume that GPs are more willing to fill in the questionnaire when it contains less questions. In addition, we have added questions about the expectations of the implementation of the NP in terms of workload, satisfaction with providing care, quality and efficiency of care, referrals and collaboration with colleagues. Questions about the collaboration with the NP have been added for the measurement at the end of the intervention period.

Since we assume that knowledge is a prerequisite for the delivery of good quality care, a knowledge assessment is used to assess the NPs. As a reference group we use a selection of GPs. The assessment include 160 questions derived from the National GP knowledge test [30]. The questions concern only health problems that, according to the MANP and their extra training, can be diagnosed and treated by NPs. The questions include true/false answers. There is also an opportunity to fill in a ‘question mark’ in the case the participant does not know the answer.

#### Medical research utilization and costs

An economic evaluation is conducted alongside the quasi-experimental study. Direct costs within the out of hours care included resource use (e.g. prescriptions, test & investigations, referral to other ED or hospital), length of consultations, type of consultations (i.e. telephonic consultation, face to face consultation or home visit), consultation rate and salary costs. Our economic evaluation is based on the general principles of a cost-minimization analysis. This choice of evaluation is based on the evidence available in the literature that shows that the introduction of the NPs comes with improved or equal quality of care, clinical outcome. So, it seems appropriate to hypothesize that clinical effectiveness is better or at least as good and that the substitution GP with NP results in a cost reduction. The unknown is, how big the cost reduction for an GPC will be in the Netherlands. Prices will be based on the Handbook for cost studies [31]. Data will be derived from the electronic medical records during the intervention period as well as from the same 15 month period in 2009–2010.

#### Feasibility, barriers and facilitators

One month before the introduction of NPs, the feasibility of allocation of care and barriers and facilitators for the implementation of an NP in out of hours care are explored by semi-structured interviews with 4 GPs, 5 practice assistants, 5 NPs and the manager and 3 physicians working at the ED. We developed an interview protocol based on the protocol used in the study to evaluate the implementation of NPs in day practices [32]. Possible solutions for the expanding workload in the acute care sector, expectations of the implementation of NPs, responsibilities considering the work of the NP and barriers and facilitators for the implementation of an NP in out of hours care are topics that are discussed. This interview will be repeated at the end month of the intervention period.

#### Statistical power

No power is calculated since the number of patients is defined by all patients consulting a professional (either NP or GP) during weekend days between 10 am and 17 pm during 15 month intervention period. Based on historical data it is expected that during these days approximately 12.750 patients will consult the GPC.

With regard to patient satisfaction a convenient sample of maximum 200 patients per professional is taken. This number is equal to CQ-index procedures [27].

With regard to subjective workload all GPs working at the GPC and practice assistants or triage nurses employed at the front office are invited to fill in the questionnaire.

### Data analysis

Descriptive analyses (e.g. percentage, mean, standard deviation, median and inter quartile ranges) will be calculated for our primary outcome (substitution of care) and secondary outcomes (patient satisfaction and subjective workload). Normally distribution of the outcomes will be assessed to determine appropriate statistical analyses; we will conduct a multivariate (regression) analysis to compare experimental and control condition, and within the experimental group the work of NPs will be compared with GPs. To determine the effect on GPs workload baseline data will be compared with post-measurement data. A 5% significant level is used to determine difference between groups.

For the analysis of quality and safety of care (the video-audio material) we calculated overall ‘adherence’ scores derived from the checklists based on the Clinical Practice Guidelines for General Practitioners and the MAAS-Global instrument. Two observers (GPs) will score the consultations independently and the inter-rater reliability will be used to determine their agreement on the scores. We will use the program Noldus Observer to analyze the video-audio recordings.

Mean costs are calculated for experimental and control condition and compared using Student *t*-test (two-sided; alpha =0.05). Based on the literature – assuming equal care provision - it is anticipated that we will apply cost-minimization analysis. In the unlikely situation that the experimental condition is more expensive than the control condition we will determine the incremental cost-effectiveness ratios (ICERs) per QALY gained (EQ-5D) and/or per patient satisfaction. The SPSS software version 18 will be used to analyze the data. P-value is set at 0.05 as the statistical significance level in each analysis.

The interviews will be qualitatively analyzed with ATLAS.ti. The interviews will be recorded and transcribed with participants consent. We will use the constant comparative method to analyze the data [33]. Firstly, codes will be given to specific text fragments. Secondly, the codes that refer to the same phenomenon are grouped into categories and those categories will be grouped into themes. Two researchers will study the transcript independently to reduce subjectivity. They will reach consensus by discussion.

## Discussion

The rising demand for acute care and the impending shortage of GPs in the future largely affects the workload of GPs. This emphasizes the need for adequate solutions to reduce this workload and maintain the accessibility, efficiency and quality of out of hours care in the future. Shifting tasks from GPs to qualified nurses is one possible solution, but the effect of substitution of care during out-of-hours-care by nurse practitioners is largely unknown. Some studies however studied the effects of the use of nurses for the telephone triage or telephone consultations [34,35]. Others, studied the effects of the use of NPs in a hospital setting [36,37] and the effectiveness of emergency care practitioners (ECP) in a primary care out of hours service [38,39]. Nevertheless, it is unknown if nurse practitioners are also useful to perform face to face consultations in a primary out of hours setting.

A strength of this study is that we collect a very large set of data from different sources which provides not only useful information for the effect evaluation but with the interviews we are also able to determine barriers and facilitators for implementation (i.e. process evaluation). This will help us in the interpretation of the results and provide possible key components for the implementation of an NP in an out of hours care setting [40]. We did not choose to use an RCT design, as that would have disturbed the normal process at the GPC too much. We do not have the opportunity to influence the patient population who consult the GP or NP. Instead GPs and NPs choose the patients out of the common diary. Personal preferences of the NPs and GPs could perhaps influence the selection of patients and cause confounding. However, the set up is very similar to daytime primary care. Both NPs and GPs see a broad spectrum of common complaints. Patient selection by NPs is in concordance with the NP training program [28]. Nevertheless, the high pragmatic attitude of this study can be seen as a strength since it fits closely to the normal procedure at the GPC and could therefore increase feasibility.

This study started in December 2010 by designing the program and making all practical arrangements for the start and implementation of the intervention. In April 2011 the intervention period started and will continue until July 2012. The next 12 months are scheduled for analysing the data and for the implementation and consolidation of the NP as a standard employee at the GPC (when outcomes are positive). The results of this study will become available mid 2013.

## Abbreviations

GP, General Practitioner; NP, Nurse Practitioner; GPC, General Practitioner Cooperative; ED, Emergency Department.

## Competing interests

The authors declare that they have no competing interest.

## Authors’ contributions

ML and RB are responsible for the design of the study with comments from LS and HV. NW and ML wrote first draft of the manuscript and all other authors revised the manuscript critically. RB was, in collaboration with NW and ML, responsible for the preparation of the NP implementation. NW is responsible for the data-collection and data management with direct supervision and feedback from ML. Comments given in the research group includes all authors. All authors read and approved the final manuscript.

## Pre-publication history

The pre-publication history for this paper can be accessed here:

http://www.biomedcentral.com/1471-2296/13/75/prepub

## Supplementary Material

Additional file 1 ** ICPC-codes per domain for nurse practitioners based on[**[41]**]and operationized for out of hours care.** Click here for file
