# Supplementary material for: The effectiveness of Nurse Practitioners working at a GP cooperative: a study protocol
Source: BMC Fam Pract. 2012 Aug 7;13:75. doi: 10.1186/1471-2296-13-75 (PMC3503817; doi:10.1186/1471-2296-13-75)
Supplement: Additional file 1 — ICPC-codes per domain for nurse practitioners based on[[41]]and operationized for out of hours care. [file 1471-2296-13-75-S1.doc]

**BOX OF APPENDIX 1 ICPC-CODES PER DOMAIN FOR NURSE PRACTITIONERS BASED ON [1] AND OPERATIONIZED FOR OUT OF HOURS CARE**

| **A: General and unspecified**  A03 Fever, A71 measles, A72 chickenpox, A74 Rubella, A76 viral exanthem other |
| --- |
| **D: Digestive**  D05 Perianal itching |
| **F: Eye**  F01 Eye pain, F02 Red eye, F03 Eye discharge, F15 Eye appearance abnormal, F16 Eyelid, symptom/complaint, F17 Glasses symptom/complaint, F18 Contact lens symptom/complaint, F70 Conjunctivitis infectious, F71 Conjunctivitis allergic, F72 Blepharitis/stye/chalazion, F73 Eye infection/inflammation other, F74 Neoplasm of eye/adnexa, F75 Contusion/haemorrhage eye, F76 Foreign body in eye |
| **H: Ear**  H01 Ear pain/earache, H04 Ear discharge, H05 Bleeding ear, H13 Plugged feeling ear, H29 Ear symptom/complaint other, H70 Otitis externa,H71 Acute otitis media/myringitis, H72 Serous otitis media, H76 Foreign body in ear, H77 Perforation ear drum, H78 Superficial injury of ear, H79 Ear injury other, H81 Excessive ear wax, H99 Ear/mastoid disease, other |
| **K: Cardiovascular**  K96 Haemorrhoids |
| **L: Musculoskeletal**  L02 Back symptom/complaint, L03 Low back symptom/complaint, L04 Chest symptom/complaint, L05 Flank/axilla symptom/complaint, L07 Jaw symptom/complaint, L08 Shoulder, symptom/complaint, L09 Arm symptom/complaint, L11 Wrist symptom/complaint, L12 Hand/finger symptom/complaint, L13 Hip symptom/complaint, L14 Leg/thigh symptom/complaint, L15 Knee symptom/complaint, L16 Ankle symptom/complaint, L17 Foot/toe symptom/complaint, L18 Muscle pain, L19 Muscle symptom/complaint NOS, L20 Joint symptom/complaint NOS, L72 Fracture: radius/ulna, L73 Fracture: tibia/fibula, L74 Fracture: hand/foot bone, L75 Fracture: femur, L76 Fracture: other, L77 Sprain/strain of ankle, L78 Sprain/strain of knee, L79 Sprain/strain of joint NOS  L80 Dislocation/subluxation, L81 Injury musculoskeletal NOS, L86 Back syndrome with radiating pain, L93 Tennis elbow |
| **N: Neurological**  N72 Tetanus |
| **R: Respiratory**  R01 Pain respiratory system, R02 Shortness of breath/dyspnoea, R03 Wheezing, R05 Cough, R06 Nose bleed/epistaxis, R07 Sneezing/nasal congestion, R08 Nose symptom/complaint other, R09 Sinus symptom/complaint, R21 Throat symptom/complaint, R23 Voice symptom/complaint, R24 Haemoptysis, R25 Sputum/phlegm abnormal, R71 Whooping cough, R72 Strep throat, R73 Boil/abscess nose, R74 Upper respiratory infection acute, R75 Sinusitis acute/chronic, R76 Tonsillitis acute, R77 Laryngitis/tracheitis acute, R78 Acute bronchitis/bronchiolitis, R80 Influenza, R81 Pneumonia, R87 Foreign body nose/larynx/bronch, R90 Hypertrophy tonsils/adenoids, R97 Allergic rhinitis, R98 Hyperventilation syndrome |
| **S: Skin**  S01 Pain/tenderness of skin, S03 Warts, S04 Lump/swelling localized, S05 Lumps/swellings generalized, S06 Rash localized, S07 Rash generalized, S08 Skin colour change  S09 Infected finger/toe, S10 Boil/carbuncle, S11 Skin infection post-traumatic, S12 Insect bite/sting  S13 Animal/human bite, S14 Burn/scald, S15 Foreign body in skin, S16 Bruise/contusion, S17 Abrasion/scratch/blister, S18 Laceration/cut, S19 Skin injury other, S20 Corn/callosity, S21 Skin texture symptom/complaint, S27 Fear of skin disease other, S28 Limited function/disability (s), S29 Skin symptom/complaint other, S70 Herpes zoster, S71 Herpes simplex, S72 Scabies/other acariasis, S73 Pediculosis/skin infestation other, S74 Dermatophytosis, S75 Moniliasis/candidiasis skin, S76 Skin infection other, S81 Haemangioma/lymphangioma, S84 Impetigo, S85 Pilonidal cyst/fistula, S86 Dermatitis seborrhoeic, S89 Diaper rash, S93 Sebaceous cyst, S94 Ingrowing nail, S95 Molluscum contagiosum, S97 Chronic ulcer skin, S98 Urticaria, S99 Skin disease, other |
| **T: Endocrine/Metabolic and Nutritional**  T92 Gout |
| **U: Urological**  U01 Dysuria/painful urination, U02 Urinary frequency/urgency, U71 Cystitis/urinary infection other |
| **W: Pregnancy, Childbearing, Family Planning**  W10 Contraception postcoital |
| **X: Female Genital**  X14 Vaginal discharge, X70 Syphilis female, X71 Gonorrhoea female, X72 Genital candidiasis female,  X73 Genital trichomoniasis female, X90 Genital herpes female, X91 Condylomata acuminata female |
| **Y: Male Genital**  Y25 Fear sexually transmitted dis. Male, Y70 Syphilis male, Y71 Gonorrhoea male, Y72 Genital herpes male, Y73 Prostatitis/seminal vesiculitis, Y74 Orchitis/epididymitis, Y75 Balanitis, Y76 Condylomata acuminata male. |

1. Dierick-van Daele AT, metsemakers JF, Derckx EW, Spreeuwenberg C, Vrijhoef HJ: **Nurse Practitioner in de huisartsenpraktijk: Onderzoeksrapport**. In*.* Maastricht: Maastricht UMC+; 2008.
